# Page Kidney: An Unusual Cause of Acute Onset Hypertension—A Case Report

**DOI:** 10.3390/reports9030273

**Published:** 2026-08-14

**Authors:** Konstantinos Koutsoulas, Evangelos Karagiannis, Dimitrios Kouroupis, Ioannis Vlachos, Spyros Papadopoulos, Panagiotis Pateinakis, Athina Pyrpasopoulou, Ioannis Vouros, Ioannis Goulis

**Affiliations:** 1 4th Department of Internal Medicine, Hippokration Hospital Thessaloniki, Konstantinoupoleos 49, 54642 Thessaloniki, Greece; kostaskoutsoul@gmail.com (K.K.); igoulis@gmail.com (I.G.); 2 Urology Department, Hippokration Hospital Thessaloniki, 54642 Thessaloniki, Greece; vgskarag@hotmail.gr (E.K.); imvouros@hotmail.com (I.V.); 3 2nd Propedeutic Department of Internal Medicine, Hippokration Hospital Thessaloniki, 54642 Thessaloniki, Greece; dimcour841@gmail.com (D.K.); pateinakis@hotmail.com (P.P.); 4 Department of Radiology, Hippokration Hospital Thessaloniki, 54642 Thessaloniki, Greece; troxojohny@gmail.com (I.V.); myspiros@yahoo.com (S.P.)

**Keywords:** page kidney, hematoma, renin-dependent hypertension

## Abstract

**Background and Clinical Significance**: Renal disease is the leading cause of secondary hypertension in children and adolescents. Among younger patients presenting with severe hypertension, renovascular and renal parenchymal disorders should be considered promptly; **Case presentation**: We describe the case of a 16-year-old male who presented with severe fatigue and was found to have resistant arterial hypertension (180/120 mmHg). His medical history was notable for blunt epigastric trauma sustained during football training approximately 6 months before presentation. Magnetic resonance imaging of the kidneys and retroperitoneum demonstrated a large right-sided perinephric hematoma compressing the kidney. Plasma renin activity and aldosterone levels were markedly elevated, establishing the diagnosis of Page kidney. Percutaneous drainage was performed by placement of a drainage catheter into the perinephric collection, resulting in evacuation of a substantial volume of liquefied hematoma. Following the procedure, arterial blood pressure gradually normalized, accompanied by resolution of the hormonal abnormalities; **Conclusions**: Page kidney is a rare but important cause of secondary hypertension resulting from activation of the renin-angiotensin-aldosterone system due to external renal compression and impaired intrarenal perfusion. Although its clinical presentation may be insidious, delayed recognition can lead to severe cardiovascular and renal complications. Management includes percutaneous drainage or surgical decortication of the affected kidney, together with antihypertensive treatment targeting the renin-angiotensin-aldosterone system. Early diagnosis and treatment are essential to optimize clinical outcomes and preserve renal function.

## 1. Introduction and Clinical Significance

According to both the European and the British Societies of Hypertension, arterial hypertension, when diagnosed in young adults/adolescents, is often secondary to underlying medical conditions and/or external factors [1]. In this age group, secondary causes are considerably more common than in older adults and include endocrine disorders—such as hypothyroidism (1.9%), primary hyperaldosteronism (1.2%), Cushing syndrome (0.5%), and pheochromocytoma (<0.3%)—as well as renal diseases, including renovascular disease (1.7%) and renal insufficiency (1.5%) [2,3]. The European Society of Hypertension (ESH) recommends referral to specialized hypertension centers for patients younger than 40 years old who present with an initial blood pressure exceeding 160/100 mmHg [4]. We report the case of a 16-year-old male who presented with severe arterial hypertension and was diagnosed with Page kidney, a rare, trauma-related, renin-dependent cause of secondary hypertension [5].

## 2. Case Presentation

### 2.1. Patient Information and Clinical Presentation

A 16-year-old male presented to the Emergency Department with severe arterial hypertension (180/120 mmHg). His personal and family medical history were unremarkable, and he was not taking any regular medications, dietary supplements, or recreational drugs. Two weeks before admission, he had been evaluated by his pediatrician and subsequently by a cardiologist because of progressive fatigue and reduced exercise tolerance during football training.

Physical examination was unremarkable apart from markedly elevated blood pressure. Laboratory investigations, including liver function tests (aspartate aminotransferase (AST) 25 U/L, normal values 10–37 U/L, and alanine aminotransferase (ALT) 14U/L, normal values 10–45 U/L), serum creatinine (0.79 mg/dL, normal values < 0.9 mg/dL), urinalysis, hemoglobin concentration (13.5 g/dL, normal values 13.5–17.5 g/dL), coagulation profile and C-reactive protein levels (5.8 mg/L, normal values < 6 mg/L), were all within the normal reference range. Transthoracic echocardiography demonstrated normal cardiac structure and function, but incidentally revealed a complex cystic lesion located in the anatomical region of the right kidney. The patient was therefore referred for further investigation. Neurological examination performed at the Emergency Department, including bedside fundoscopy, did not reveal papillary edema or any other significant abnormalities.

### 2.2. Laboratory and Radiological Investigation

**Figure 1:**
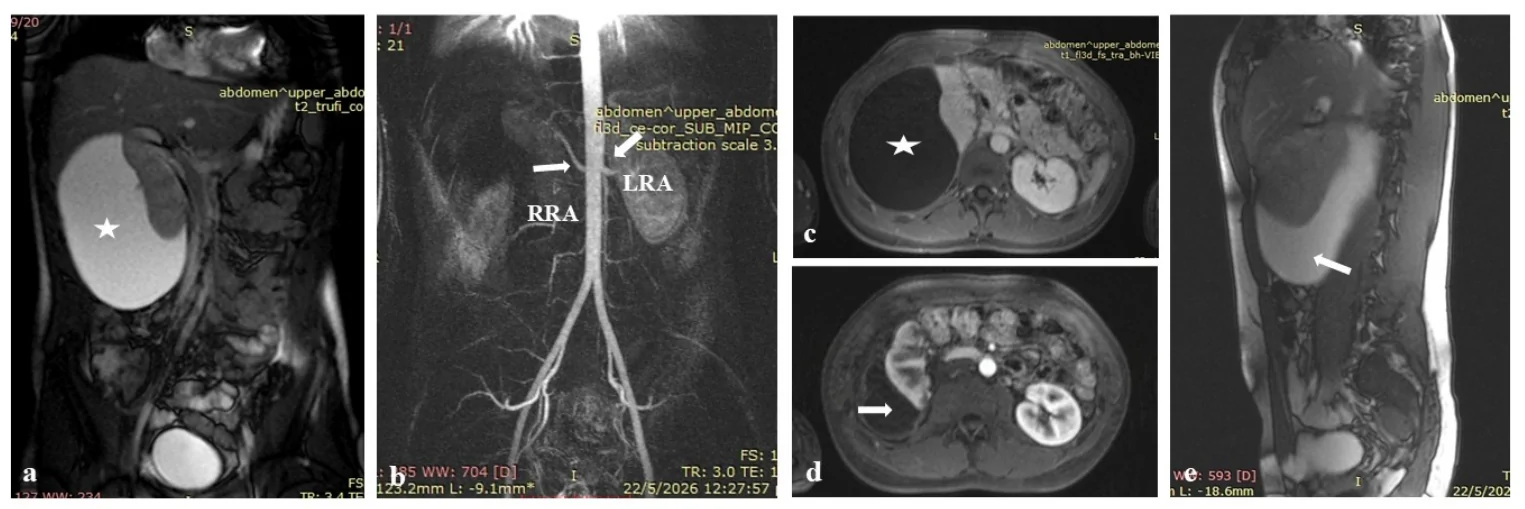
MR imaging of the patient’s upper abdomen/retroperitoneum revealing a significant fluid collection in the perinephric region of the right kidney consistent with a chronic liquefied hematoma (**a**–**e**). The collection severely compresses the parenchyma of the right kidney. (**a**,**e**) Sagittal magnetic resonance (MR) imaging planes of the upper abdomen/retroperitoneum before (**a**) and after (**e**) percutaneous drainage with significant decrease in the size of the collection post-drainage (white star and white arrow respectively) (**b**). Renal artery MR angiography (MRA) providing imaging of the aorta and renal arteries (arrows) without any significant stenoses of the renal artery stumps. Arterial dissection and active bleeding were ruled out (**a**,**e**). (**c**,**d**) Corresponding tangential MR imaging planes of the kidneys after nephrostomy catheter insertion and drainage of 1700 mL of the serous collection underneath the renal (Gerota) fascia ((**c**): before drainage indicated with a star, (**d**): after drainage indicated with an arrow). RRA: Right Renal Artery; LRA: Left Renal Artery.

On admission, arterial hypertension was the only remarkable clinical feature (158/96 mmHg after the administration of 5 mg amlodipine at the Emergency Department). Repeat laboratory investigations, urinalysis, electrocardiography and chest radiography were unremarkable. Plasma renin activity and serum aldosterone levels, obtained before initiation of antihypertensive therapy, were markedly elevated (plasma renin activity (PRA) 9.261 ng/mL/hour, reference range 0.32–1.84 ng/mL/hour; serum aldosterone 800.1 pg/mL, reference range 68–173 pg/mL). No abdominal or flank bruits were detected. Given the incidental ultrasonographic findings, magnetic resonance imaging (MRI) of the kidneys and retroperitoneum, together with magnetic resonance angiography (MRA), was performed. They demonstrated a large right-sided perinephric hematoma with liquefaction and internal septations causing marked compression and peripheral displacement of the renal parenchyma (Figure 1a–c). Following a detailed review of his medical history, the patient recalled sustaining blunt epigastric trauma during football training approximately 6 months earlier, followed by intermittent mild discomfort in the affected area. As the symptoms had been minimal, no medical investigation had been sought at that time.

### 2.3. Diagnosis and Management

Based on the combination of severe hypertension, elevated renin activity and aldosterone levels, previous blunt abdominal trauma, and imaging evidence of significant external compression of the right kidney by a chronic hematoma, the diagnosis of Page kidney was established [6]. A drainage catheter was inserted percutaneously by the urology team under ultrasound guidance into the perinephric collection. A total of 1700 mL of serous fluid was drained (1200 mL immediately after catheter placement and an additional 500 mL following catheter repositioning three days later). Surgical decortication was considered a rescue treatment in the case in which percutaneous drainage had not been successful.

Repeat MRI demonstrated satisfactory re-expansion of the renal parenchyma (Figure 1d,e) and the drainage catheter was removed prior to discharge eight days later. The patient was scheduled for outpatient follow-up with serial blood pressure measurements and repeat renal ultrasonography.

### 2.4. Patient Follow-Up and Outcome

**Figure 2:**
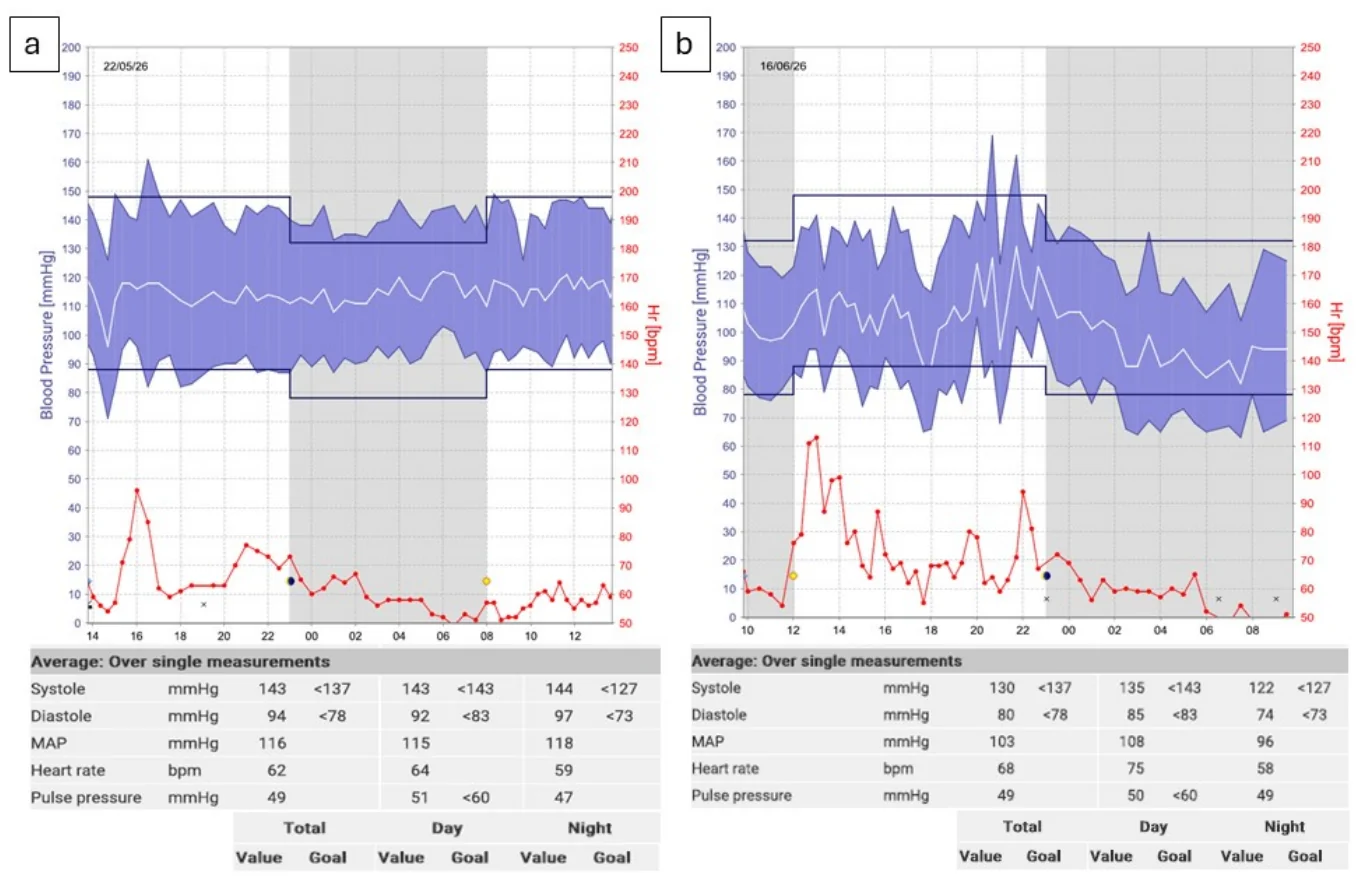
The 24 h blood pressure measurement (**a**) immediately after percutaneous drainage and decompression of the right kidney and (**b**) repeat measurement three weeks later. The black and yellow dots (•) indicate the beginning of night-time and day-time blood pressure measurements respectively.

Blood pressure progressively declined following renal decompression, with a mean arterial pressure of 116 mmHg immediately after the procedure (Figure 2a). Because of the rapid clinical improvement, the patient was discharged without regular antihypertensive therapy. His family was instructed to monitor his blood pressure regularly and administer amlodipine only if systolic blood pressure exceeded 140 mmHg. Three weeks after discharge, 24 h ambulatory blood pressure demonstrated a mean arterial pressure of 103 mmHg (Figure 2b). PRA and serum aldosterone concentrations had returned to within the normal reference range (0.83 ng/mL/hour and 87.0 pg/mL respectively). The patient will be re-evaluated for potential dynamic renal scintigraphy to evaluate renal perfusion and excretory function [7].

## 3. Discussion

### 3.1. Pathophysiology and Clinical Presentation of Page Kidney

Page kidney was first described in 1939 by Dr Irvine Page who induced persistent hypertension in experimental animals by wrapping canine kidneys in cellophane, thereby eliciting an inflammatory reaction and compressing the parenchyma and its intrarenal microvasculature. The resulting microvascular ischemia activates the renin–angiotensin–aldosterone system (RAAS), leading to renin-dependent hypertension [6,8,9]. The concept that localized renal ischemia alone is sufficient to induce sustained hypertension had previously been demonstrated in experimental animal models [10]. A similar pathophysiological mechanism has also been reported following renal compression caused by abdominal packing during damage control surgery for severe intra-abdominal hemorrhage [11].

The degree of renal functional impairment depends on the severity and duration of main renal artery compression. In clinical practice, Page kidney most commonly develops secondary to external compression of the renal parenchyma by a subcapsular or perinephric fluid collection, most frequently a post-traumatic hematoma, as observed in our patient. Young males are most commonly affected [12].

Hypertension is the hallmark clinical manifestation and may develop immediately after the inciting event or remain clinically silent for months or even years. Other reported manifestations include, in order of decreasing frequency, flank pain, hematuria, abdominal pain, and back pain. Our patient presented with only mild, non-specific symptoms and had no laboratory evidence suggestive of kidney injury to prompt further investigation.

### 3.2. Etiology of Page Kidney

Blood or fluid may accumulate within two distinct spaces surrounding the kidney. Smaller collections develop beneath the renal capsule (subcapsular), whereas larger collections accumulate within the perirenal space enclosed by Gerota’s fascia, as occurred in our patient [6]. Because Gerota’s fascia provides a larger potential space for fluid accumulation, substantially greater fluid volumes are generally required before clinically significant compression of the renal parenchyma occurs.

The timing of diagnosis is strongly influenced by the location of the collection. Subcapsular hematomas produce high-pressure compression of the renal parenchyma and therefore typically present earlier. In contrast, more commonly, perirenal (extracapsular) collections usually develop more gradually and often present months after the initial insult. Consistent with the later mechanism, our patient developed hypertension approximately six months after the traumatic event.

Page kidney remains a rare clinical entity. Historically, the typical patient was a young, otherwise healthy, male athlete presenting with hypertension following blunt sport-related trauma, similar to the present case. It has, however, been described in all age groups. More recently, an increasing number of non-traumatic cases have been described, broadening the spectrum of recognized etiologies [6,13]. In reviews and large case series, causes leading to its development have been classified as bleeding (trauma-related, iatrogenic, spontaneous bleeding and secondary to cyst ruptures), and non-bleeding (spontaneous and idiopathic), summarized in Table 1, with small differences regarding localization.

Since the early 1990s, published case series have demonstrated a shift toward predominantly iatrogenic causes, probably reflecting the increasing number of diagnostic and therapeutic renal interventions performed in modern clinical practice. Most iatrogenic cases occur following renal biopsy, extracorporeal shock wave lithotripsy, ureteronephroscopy or renal transplantation and are usually associated with subcapsular hematomas.

### 3.3. Management of Page Kidney

The primary goal of treatment is prompt relief of external renal compression to restore renal perfusion, preserve renal function, and reverse RAAS activation. Whenever feasible, minimally invasive techniques are preferred, including percutaneous catheter drainage or surgical capsulotomy [17]. Because chronic hematomas frequently contain highly viscous or partially liquefied material, simple needle aspiration is often ineffective. Placement of a pigtail drainage catheter using the Seldinger technique under ultrasonographic guidance provides more effective decompression [18]. Preservation of renal function is particularly important in patients with solitary kidneys or transplanted renal allografts, in whom nephron loss may have substantial clinical consequences [17]. Less invasive and aggressive treatment choices are therefore preferred, whenever technically feasible. Based on the chronicity of the hematoma in our patient and its morphologic characteristics, percutaneous drainage with ultrasonographic guidance was attempted successfully. Failure to efficiently drain the collection and decompress the kidney would have inevitably led to surgical renal decortication. In a large systematic review through March 2023 including 24 cases in total, delayed diagnosis was significantly associated with the need for more invasive surgical procedures (i.e., nephrectomy) [17]. On the other hand, kidney preservation was more often observed when diagnosis was made within days to months (with a median time to diagnosis of 8 to 22 days). Our patient’s renal function was preserved at diagnosis, despite his delayed presentation. This was probably due to young age, absence of pre-existing vascular disease, and his otherwise healthy renal parenchyma. Progressive elevation of blood pressure reflects sustained compression of the renal vasculature and persistent activation of the RAAS, which, if untreated, may ultimately result in irreversible renal injury. Encouragingly, blood pressure typically normalizes following successful renal decompression, as observed in our patient [19].

Acute Page kidney caused by rupture of renal arterial aneurysms or vascular malformations may require alternative interventions, including selective arterial embolization [20]. When definitive decompression cannot be performed immediately or conservative management is selected, RAAS blockade constitutes the cornerstone of antihypertensive therapy [21]. A retrospective study comparing traumatic and non-traumatic Page kidney cases found that patients with traumatic disease were generally younger, had less severe hypertension, and exhibited better preserved renal function at presentation, whereas imaging findings, urinalysis, and pathological characteristics were similar between the two groups [13]. In our case, the patient required only temporary antihypertensive therapy with amlodipine on an as-needed basis after discharge. Ambulatory blood pressure monitoring performed three weeks later demonstrated complete normalization of blood pressure, and no antihypertensive medication was ultimately required.

## 4. Conclusions

Page kidney is an uncommon but important cause of secondary, renin-dependent hypertension resulting from external compression of the renal parenchyma and subsequent activation of the renin–angiotensin–aldosterone system. Although the condition may develop insidiously, it has the potential to present as a hypertensive emergency or lead to irreversible renal damage if diagnosis is delayed. Our patient developed clinically significant hypertension six months after sustaining blunt abdominal trauma, despite having normal laboratory findings and only subtle symptoms. Diagnosis was ultimately prompted by abnormal renal imaging, highlighting the importance of maintaining a high index of suspicion and obtaining a thorough history in young patients presenting with severe or resistant hypertension.

Early recognition of Page kidney allows for timely decompression of the affected kidney, promotes normalization of blood pressure, preserves renal function, and reduces the need for more invasive surgical intervention. Secondary causes of hypertension should always be considered in patients with new-onset severe hypertension, especially children, adolescents, and young adults. Prompt diagnosis is associated with favorable outcome and preserves renal function.

## Figures and Tables

**Table 1:** Classification of causes leading to compression of the renal parenchyma and their major features [5,12,13,14,15,16].

Cause	Related to	Localization	Unilateral/Bilateral	Treatment	Prognosis
Bleeding Causes (accumulation of hemorrhagic material)					
Blunt Trauma (Classic Page Kidney)	Sport injuries like football, hockey, other contact sports, and motor vehicle accidents, violence, or falls	Extracapsular/perinephric	Unilateral (extremely rarely bilateral)	Surgical ± medical	Good
Iatrogenic	Biopsy of native or transplant kidney, extracorporeal shockwave lithotripsy, ureteral surgical procedure, sympathetic nerve block	Subcapsular	Unilateral	Surgical ± medical	Variable
Spontaneous Bleeding (Including Wunderlich Syndrome)	Arteriovenous malformations, underlying tumors, cyst rupture, pyelonephritis, vasculitis/glomerulonephritis, pregnancy	Subcapsular/perinephric	Unilateral/Bilateral	Surgical/ Arterial embolization	Good (worse in the case of anticoagulation)
Cyst Rupture	Usually in patients with polycystic disease	Subcapsular	Unilateral	Surgical	Good (worse in the case of anticoagulation)
Non-Bleeding Causes (non-hemorrhagic fluid collections)					
Spontaneous Non-Bleeding	Lymphatic collections and subcapsular urinomas	Subcapsular/perinephric	Unilateral/Bilateral	Conservative management to interventional approaches such as percutaneous aspiration, sclerotherapy, surgical cyst deroofing in symptomatic/recurrent cases	Variable
Idiopathic	Nephrotic syndrome, Eisenmenger syndrome, undefined cause	Perinephric	Unilateral/Bilateral	Surgical	Good

## Data Availability

The data underlying this case report are not publicly available because they contain information that could compromise patient privacy. De-identified details may be available from the corresponding author on reasonable request and subject to institutional approval.
